# Editorial: Five-membered ring heterocyclic compounds as anticancer drug candidates

**DOI:** 10.3389/fchem.2025.1599140

**Published:** 2025-04-02

**Authors:** Arif Mermer, İlkay Erdogan Orhan, Gang Ye, Nv Anil Kumar, Ramona Danac

**Affiliations:** ^1^ Faculty of Pharmacy, University of Health Sciences, İstanbul, Türkiye; ^2^ Experimental Medicine Application and Research Center, Validebag Research Park, University of Health Sciences, İstanbul, Türkiye; ^3^ Department of Biotechnology, Hamidiye Institute of Health Sciences, University of Health Sciences, İstanbul, Türkiye; ^4^ Department of Pharmacognosy, Faculty of Pharmacy, Lokman Hekim University, Ankara, Türkiye; ^5^ Natural Medicine Research Center, College of Veterinary Medicine, Sichuan Agricultural University, Chengdu, China; ^6^ Department of Chemistry, Manipal Institute of Technology, Manipal Academy of Higher Education, Manipal, India; ^7^ Chemistry Department, Faculty of Chemistry, Alexandru Ioan Cuza University of Iasi, Iasi, Romania

**Keywords:** heterocycles, anticancer, azoles, five-membered, drug discovery

The search for novel and effective anticancer agents remains a critical focus in medicinal chemistry. Among the promising candidates, five-membered ring heterocyclic compounds have attracted significant attention due to their unique structural features and diverse biological activities. The current Research Topic of *Frontiers in Chemistry* aims to explore the role of these compounds as potential anticancer drug candidates, highlighting recent advances, emerging trends, and the challenges involved in translating these compounds from the laboratory-scale studies to clinical applications.

Five-membered ring heterocycles, such as pyrroles, imidazoles, pyrazoles, thiazoles, triazoles and others, have shown potential in a variety of biological activities, including anticancer, antimicrobial, anti-inflammatory, and antioxidant properties (Kumar et al., 2023; Haider et al., 2022). Their ability to interact with numerous molecular targets such as DNA, protein kinases, and enzymes—positions them as valuable scaffolds for developing novel anticancer therapies (Kabir and Uzzaman, 2022; Rana et al., 2023).

In this Research Topic, we bring together a collection of articles received from China, Germany, Egypt, Saudi Arabia, South Africa, and United Kingdom, that provide versatile insights into the synthesis, mechanisms of action, and therapeutic potential of five-membered ring heterocycles (https://www.frontiersin.org/research-topics/63411/five-membered-ring-heterocyclic-compounds-as-anticancer-drug-candidates).

Hu et al. reported the anticancer effects of gefitinib-1,2,3-triazole derivatives on HeLa cells. One of the compounds demonstrated superior anticancer activity with an IC_50_ of 5.66 ± 0.35 μM compared to gefitinib (IC_50_ = 14.18 ± 3.19 μM) used as the reference drug. This compound also inhibited colony formation in a concentration-dependent manner, induced cell apoptosis, and arrested the cell cycle at the G2/M phase. The Western blot analysis revealed the involvement of the mitochondrial pathway, with upregulation of the Bax/Bcl-2 ratio and increased levels of active caspase-3 and PARP1 cleavage.

Ahmed et al. focused on the design, synthesis, and biological evaluation of benzimidazole/1,2,3-triazole hybrids as apoptotic antiproliferative agents targeting the EGFR pathway. Among the obtained hybrid compounds, two of them exhibited remarkable potency with GI_50_ values of 29 nM and 25 nM. These compounds not only displayed a superior EGFR inhibition compared to that of erlotinib but also promoted apoptosis through the activation of caspases-3 and -8, and the pro-apoptotic protein Bax, while down-regulating the anti-apoptotic protein Bcl-2. Furthermore, *in silico* molecular docking calculations confirmed the binding interactions of the most promising compounds with the EGFR active site, further validating their potential as EGFR inhibitors.

A novel approach for the synthesis of hybrid spirooxindoles incorporating triazolyl-s-triazine scaffolds via a [3 + 2] cycloaddition reaction of azomethine ylide (AY) with chalcones was investigated by Shawish et al. The obtained compounds were tested for their potential cytotoxicity against HepG2 and MDA-MB-231 cell lines The results showed that two chalcone derivatives exhibited the highest activity, with IC_50_ values of 7.2 ± 0.56 µM and 7.5 ± 0.281 µM. The synthesized spirooxindole derivatives also showed promising efficacy but lower than the chalcones with IC_50_ values ranging from 13.5 ± 0.92 µM to 31.3 ± 0.86 µM against HepG2 and MDA-MB-231 cell lines. Notably, one of the chalcone derivatives demonstrated better activity than the positive control Sorafenib against MDA-MB-231 cells. Molecular docking, ADMET, and molecular dynamics simulations of promising compounds, revealed strong binding affinity in the EGFR active site, providing further rationale for their anticancer potential.

Mohlala et al. reviewed on the significance of multicomponent reactions (MCRs) in medicinal chemistry and their application to the synthesis of complex organic compounds. MCRs such as the Ugi, Passerini, Biginelli, and Hantzsch reactions are recognized for their ability to rapidly generate molecular diversity. The review emphasized the use of selective MCRs in producing organic compounds with potential anticancer activity, along with their advantages in sustainable and efficient synthesis. The discussion highlights how MCRs can enhance atom economy, reduce waste, and simplify the synthesis process, making them an attractive strategy for drug discovery and development.

Indole derivatives as dual inhibitors of aromatase and inducible nitric oxide synthase (iNOS), with antiproliferative activity against various cancer cell lines were designed and synthesized by Al-Wahaibi et al. The synthesized compounds fall into two categories that are indole-2-carboxamide derivatives and pyrazino [1,2-a]indol-1(2H)-ones. The pyrazino [1,2-a]indol-1(2H)-one derivatives were found to be more potent with GI_50_ values in the nanomolar range (25–72 nM). Two of the compounds promoted apoptosis by activating caspase-3, caspase-8, and Bax, and down-regulating the anti-apoptotic protein Bcl-2. Molecular docking studies confirmed the strong binding affinity of these compounds for the aromatase active site, while ADME studies emphasized their potential as therapeutic agents with reduced toxicity.

Amino acid-derived quaternary ammonium salts were effectively used in the asymmetric aza-Henry reaction of nitromethane with N-Boc trifluoromethyl ketimines by Ren et al. The resulting α-trifluoromethyl β-nitroamines were synthesized with good to excellent yields and moderate to good enantioselectivities. The reaction was characterized by mild conditions, low catalyst loading (1 mol%) and a catalytic base, and was successfully scaled up to grams without losing enantioselectivity. The products were then converted into a series of adamantane-type compounds with chiral trifluoromethylamine fragments. The anticancer activities of the synthesized compounds against liver cancer (HepG2) and melanoma (B16F10) were evaluated, identifying six promising compounds with significant activity that have potential for further development with IC_50_ values ranging from 5.0 µM to 12.4 µM.

In conclusion, the articles in this Research Topic collectively highlight the diverse and promising potential of five-membered ring heterocyclic compounds in anticancer drug discovery. The innovative approaches, from the design of novel chemical scaffolds to the exploration of multicomponent reactions, contribute significantly to the expanding pool of anticancer candidates. As the field continues to evolve, the insights provided in this Research Topic serve as an invaluable resource for researchers and clinicians working toward the development of more effective and targeted cancer treatments.
